# Prediction of spontaneous closure of isolated ventricular septal defects in utero and postnatal life

**DOI:** 10.1186/s12887-016-0735-2

**Published:** 2016-12-08

**Authors:** Xing Li, Gui-Xian Song, Li-Jie Wu, Yu-Mei Chen, Yi Fan, Yun Wu, Ya-Hui Shen, Li Cao, Ling-Mei Qian

**Affiliations:** 1 Department of Cardiology, Wuxi Second Hospital, Nanjing Medical University, No.68 Zhongshan Road, Wuxi, Jiangsu Province China; 2 Department of Cardiology, The First Affiliated Hospital of Nanjing Medical University, No. 300 Guangzhou Road, Nanjing, 210029 Jiangsu Province China; 3 Department of Fetal Echocardiography, Nanjing Maternity and Child Health Care Hospital Affiliated to Nanjing Medical University, No.123 Tianfei Lane, Mochou Road, Nanjing, Jiangsu Province China; 4 Department of Cardiology, Jiangsu Taizhou People’s Hospital, Taizhou, Jiangsu Province China

**Keywords:** Ventricular septal defects, Spontaneous closure, Fetal echocardiography

## Abstract

**Background:**

Ventricular septal defect (VSD) is a highly prevalent fetal congenital heart defect, which can become spontaneously closed during infancy. The current study aims to characterize fetal VSDs that were subsequently spontaneously closed in the first 2 years of life in eastern China.

**Methods:**

Between January 2011 and December 2013, 257 fetal patients diagnosed with isolated VSD by fetal echocardiography at Nanjing Maternity and Child Health Care Hospital, China, were enrolled in the study. Subjects were divided into three groups: group 1 = persistent VSD; group 2 = closed after birth; group 3 = closed during gestation. Fetal echocardiography data, physical features at birth and follow-up outcomes for 2 years were compared to identify factors contributing to spontaneous closure (SC) of VSD. A predictive formula was applied to patients admitted to hospital in the first quarter of 2014 (*n* = 23) for validation.

**Results:**

SC occurred in 42.8% patients. Birth weight (3.095 ± 0.774, 3.174 ± 0.535, 3.499 ± 0.532 kg in groups 1, 2 and 3, respectively) and defect diameter (3.422 ± 0.972, 2.426 ± 0.599, 2.292 ± 0.479 mm, in groups 1, 2 and 3, respectively) showed statistically significant differences between the three groups (*P* = 0.004 and *P* = 0.000, respectively). Receiver operating characteristic (ROC) curves identified cut-off value for the defect diameter as 2.55 mm, and logistic regression analysis identified the SC probability = (1 + exp -[-2.151 − 0.716*birth weight + 1.393*diameter])^-1^. Results indicated that male fetuses, full-term birth, muscular VSD, and defects without blood flow crossing the septum, have higher incidence of SC.

**Conclusions:**

The major determinants of SC of isolated VSD are birth weight and diameter of the defect. In addition, VSD location may also affect the SC incidence.

## Background

The incidence of congenital heart disease (CHD) in the western world is 0.3–1.2% of live births [1]. Ventricular septal defect (VSD) is the most common congenital heart defect, accounting for 20–30% of all congenital heart defects [2]. In a recent study, Zhao et al. [3] described a 2.66% prevalence of CHD at live birth in China using echocardiographic screening, and they also found that only a 1.21% prevalence of CHD that could be detected by clinical evaluation, with the most common CHD as VSD at a prevalence of 1.73%. VSDs can occur alone or in combination with other cardiac defects. However, the majority of VSDs occur alone and referred to as “isolated” or “simple” [4], and the current study has therefore focused on isolated VSDs. VSDs are typically asymptomatic and often close spontaneously [5]. Two-dimensional echocardiography combined with pulsed Doppler or Doppler color flow mapping is a well-established technique to diagnose and localize fetal and infant VSDs. The present study utilized fetal echocardiography and state at birth to evaluate the incidence and timing of SC of VSD in patients who were diagnosed with VSD prenatally in eastern China. There have been a number of observational long-term follow-up studies on spontaneous closure (SC) of VSD [6–29], but few of these reports were followed up from the fetal period. Here, we present a long-term follow-up study in which subjects were followed up from the fetal period and summarized a prediction formula including factors influencing SC of VSD. We found that birth weight was a prognostic variable for SC of VSD, which was most useful for pediatricians, and that the defect diameter was another prognostic variable, which may provide useful information for obstetrician-gynaecologists to aid prenatal counseling.

## Methods

### Study population

A total of 5855 pregnant women underwent fetal echocardiography examination between January 2011 to December 2013 at the Fetal Echocardiography Department of Nanjing Maternity and Child Health Care Hospital, Nanjing, China. Of these women, 1168 cases had cardiac structural abnormalities, and 335 fetal patients diagnosed with isolated VSD were enrolled in current study.

### Indications for fetal echocardiography

Pregnant women in the following status during their routine obstetric examination were recommended a further fetal echocardiography examination: suspected CHD on screening ultrasound during the second trimester; extracardiac anomalies on screening ultrasound; advanced maternal age; family history of CHD; increased nuchal translucency; fetal arrhythmia; chromosomal abnormality.

### Diagnostic criteria of VSD by echocardiography

**Fig. 1:**
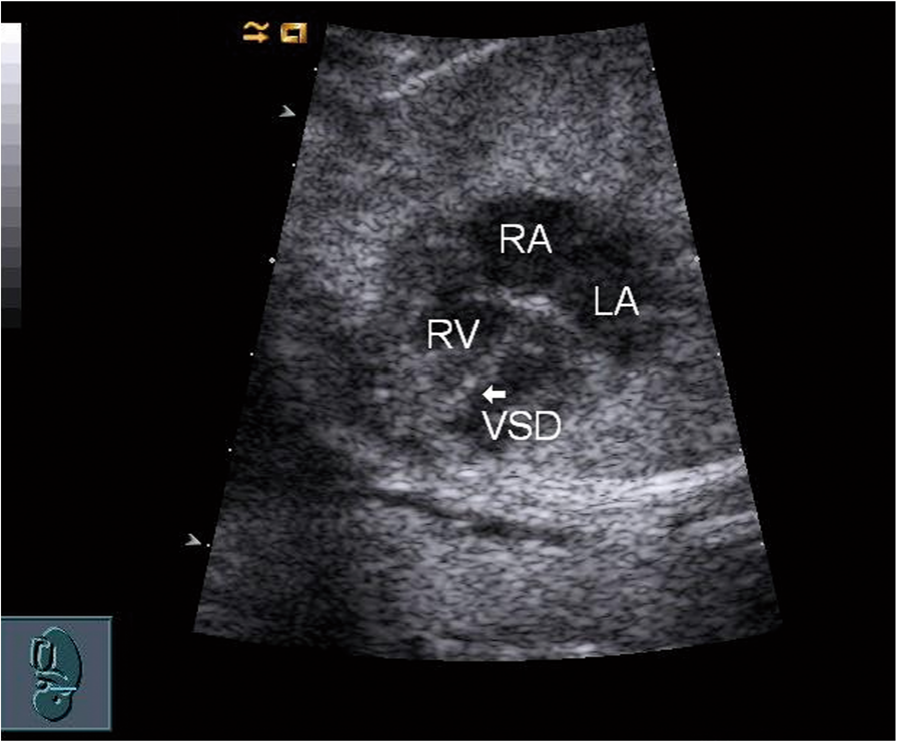
*White arrow* indicates a small VSD detected by fetal echocardiography, and SC during follow-up

**Fig. 2:**
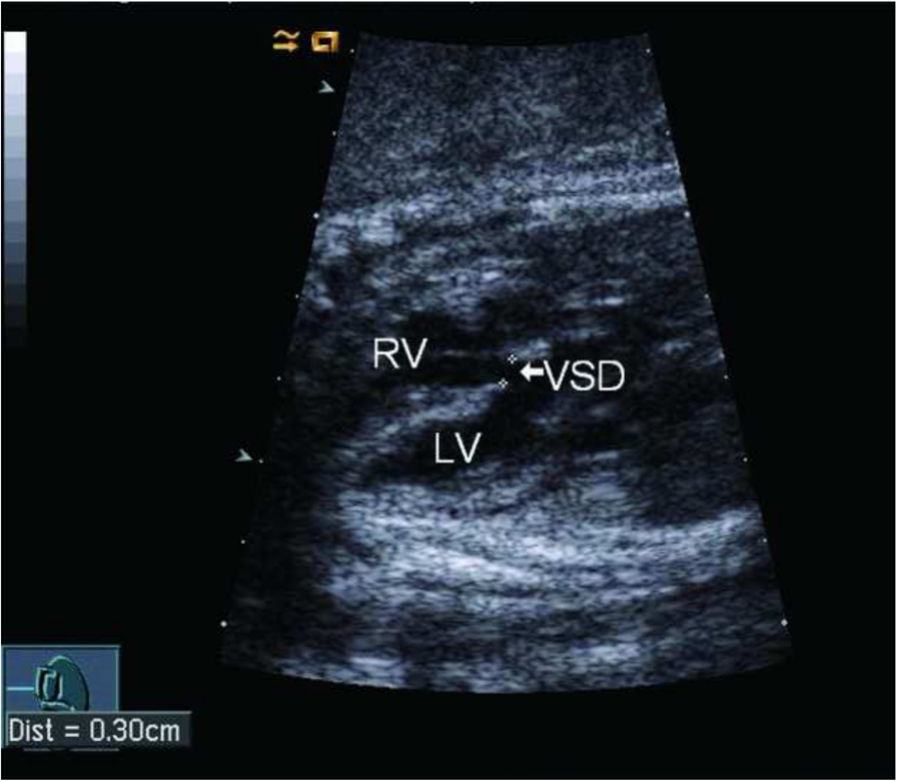
*White arrow* indicates a large VSD detected by fetal echocardiography, and no SC during follow-up

**Fig. 3:**
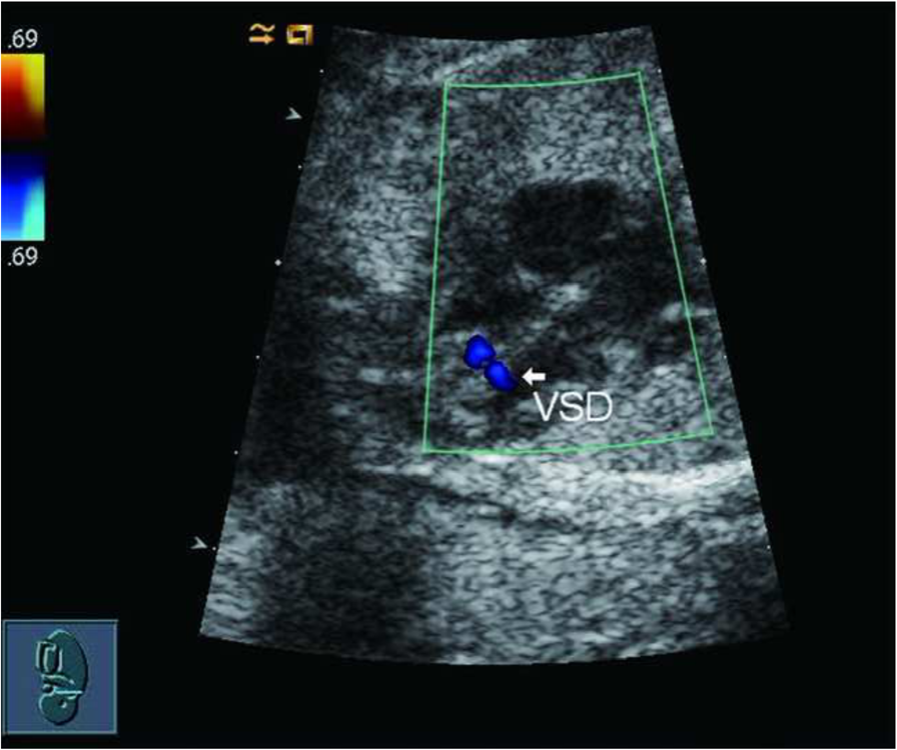
Color Doppler echocardiographic examination of small VSD

**Fig. 4:**
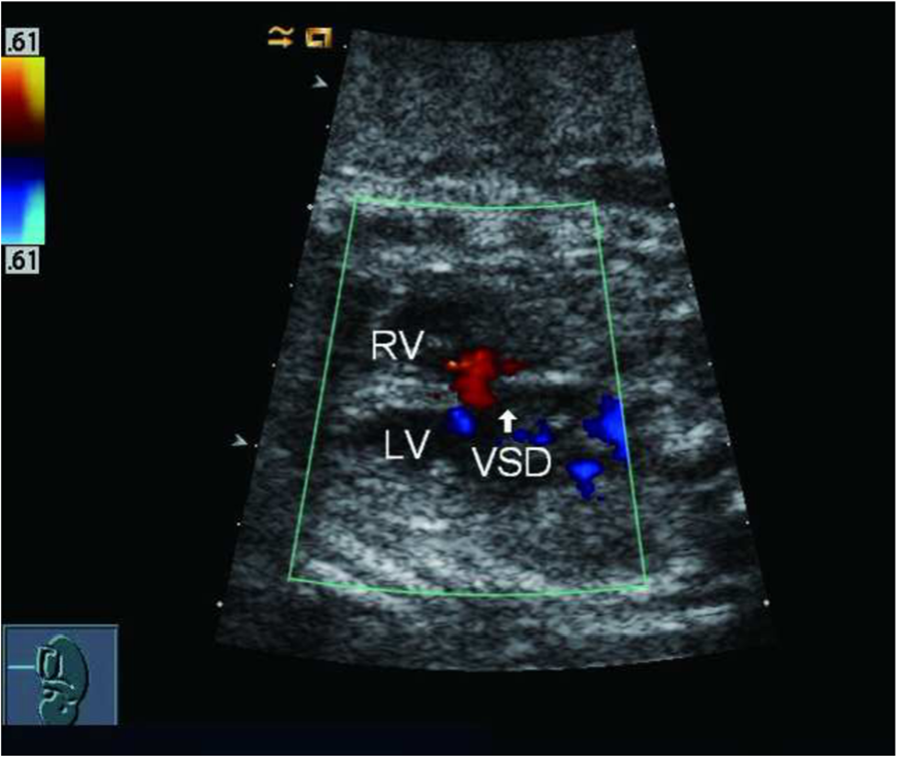
Color Doppler echocardiographic examination of large VSD

Two skilled sonographers (Li Cao and Yun Wu) conducted all the fetal echocardiography examinations using the Acuson Sequoia 512 ultrasound system (Siemens, Malvern, PA, USA) with a 3 − 5-MHz phased-array transducer. Complete transthoracic two-dimensional, M-mode, continuous wave and pulsed wave Doppler and color Doppler echocardiographic examinations were performed. An echo dropout in the ventricular septum in two-dimensional echocardiography was considered diagnostic of a VSD (Figs. 1 and 2). A typical systolic colored flame (Figs. 3 and 4) crossing the septum and in most cases a jet derived from pulsed Doppler confirmed the existence of the defect (however, if the shunt blood pressure is low, colored jet crossing the septum may not be detected). Extreme care was taken to determine location and width of defects. The diameter of VSD in two-dimensional and/or color flow mapping was measured in all planes and the largest diameter of VSD was recorded. Apical four-chamber, apical five-chamber, parasternal long- and short-axis and subcostal positions were used to image the defect, with a minimum of no less than three views. Defects were classified as perimembranous, muscular, subarterial or multiple types (more than one defect) [30]. Infants who had associated complex structural cardiovascular defects were excluded from the isolated VSD group.

### Follow-up studies

Parental consents were obtained from the parents of each neonate. Detailed history of the neonates, parents and family members were taken by face-to-face or telephone interviewing of the parents. A fetal echocardiography reexamination in third trimester and repeated echocardiographies at the children’s routine physical examinations at age 1, 3, 6 and 12 months of age depending on the growth and development condition of the children until the spontaneous or surgical closure of the VSD was confirmed, were recommended to all parents. Spontaneous closure occurred in patients without echo dropout in the septum and without detection of blood flow through the septum in echocardiography.

Data in the ultrasound records consisted of gestational weeks, maternal age, fetal heart rate, width of aorta and pulmonary artery, location and diameter of the defect, direction of the shunt. Questions in the interview included: (1) Did the defects close or not? (2) When did the spontaneous closure occur? (3) What treatments are used for those whose defects persist? (4) Gender and birth weight of the infancy. (5)Was there premature delivery? (6) Were there any infections or metabolic diseases during pregnancy? (7) Is there any family history of heart disease? Besides, fetal karyotype examination results of those who received amniocentesis were also collected.

### Validation study of the prediction formula

In the second part of the study, the prediction formula was then applied to 23 patients, who were admitted to the hospital in the first quarter of 2014 in order to validate the derived prediction formula.

### Statistical analysis

All data are expressed as mean ± standard deviation (SD). The differences among the three groups were assessed using analysis of variance (ANOVA) for continuous variables, and student-neuman-keuls tests were applied for post-hoc comparisons. Comparisons between group 1, 2 and 3 were made using Chi-square tests for categorical variables. A *P*-value <0.05 was considered statistically significant. Statistically significant clinical and echocardiographic variables were subsequently evaluated by binary logistic regression analysis to identify independent predictors of outcome. Receiver operating characteristic (ROC) analysis was performed to assess the sensitivity and specificity of the diameter of the defect for predicting SC of VSD.

## Results

**Fig. 5:**
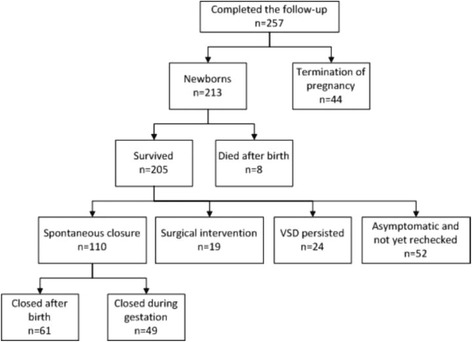
Study flow chart for the outcome of fetuses with VSD after birth

A total of 335 fetal patients, referred for fetal echocardiographic examination and diagnosed with isolated VSD at Nanjing Maternity and Child Health Care Hospital between January 2011 and December 2013, were enrolled in this retrospective study. In spite of repeated efforts to contact the parents, 78 patients were lost to follow-up after the initial first or second visit. Follow-up was completed in 257 (76.7%) patients. Outcomes for these patients are summarized in Fig. 5. According to whether the defect in ventricular septum was closed or not, children were divided into three groups: groups 1 = defects not yet closed (this group included infants who died after birth because of VSD, and who underwent surgical intervention, and whose defects persisted by the time of follow up); group 2 = defects closed after birth; group 3 = defects were closed during gestation.

### General conditions

Mean gestational age was 26.68 ± 1.092 weeks (range, 19–30 weeks) at the time when the fetuses were diagnosed with VSD. The average maternal age of the study population was 29.25 ± 4.874 years (range, 20 − 42 years). Sex ratio for the patients was 82 males: 69 females. Percentages of different types of defects were as follows: perimembranous, 115 (78.8%); muscular, 20 (13.7%); subarterial, 7 (4.8%); multiple, 4 (2.7%). The average time when spontaneous closure occurred was 7.31 ± 6.073 months.

### Clinical findings

The birth weights and diameter of the defects between the three groups were significant different (*P* = 0.004 and *P* = 0.000, respectively), with the highest birth weights observed in patients whose defects closed within gestation and the largest diameter of the defectsin patients whose defects persisted (Table 1). The proportion of preterm birth was lower in group 3 (*P* = 0.058; Table 2). Furthermore, gender differences were noted between the groups, whereby male predominance was observed in group 3 (SC during gestation) and female predominance in group 1 (defects not closed), although these differences did not reach statistical significance (Table 2). SC rates at different locations are also detailed in Table 2. All the muscular defects have been closed, higher than rate of spontaneous closure (81.8–89% at 12-month follow-up) of muscular VSD was reported in previous studies. There were 11 cases without detection of blood flow through the septum that experienced spontaneous closure.

**Table 1:** Statistical results of quantitative data are shown in Table 1

		Group 1	Group 2	Group 3	*P* value
Birth weight	M ± SD	3.095 ± 0.774	3.174 ± 0.535	3.499 ± 0.532	0.004*
	95% CI	(2.851,3.339)	(3.035,3.313)	(3.155,3.359)	
Diameter	M ± SD	3.422 ± 0.972	2.426 ± 0.599	2.292 ± 0.479	0.000*
	95%CI	(3.146,3.698)	(2.273,2.579)	(2.154,2.429)	
Maternal age	M ± SD	29.10 ± 4.610	28.43 ± 5.393	30.45 ± 4.282	0.245
Aortic diameter	M ± SD	4.62 ± 0.585	4.74 ± 0.529	4.76 ± 0.437	0.193
Pulmonary artery diameter	M ± SD	5.52 ± 0.542	5.66 ± 0.561	5.60 ± 0.481	0.469
Fetal heart rate	M ± SD	145.65 ± 8.089	146.53 ± 6.427	146.92 ± 7.957	0.177

**Table 2:** Statistical results of qualitative data are shown in Table 2

		Group 1	Group 2	Group3	*P* value
		n(%)	n(%)	n(%)	
Delivery	Premature	10/23 (43.5%)	10/23 (43.5%)	3/23 (13.0%)	0.058
	Mature	32/127 (25.2%)	51/127 (40.2%)	44/127 (34.6%)	
Gender	Male	18/82 (22.0%)	33/82 (40.2%)	31/82 (37.8%)	0.119
	Female	24/69 (34.8%)	28/69 (40.6%)	17/69 (24.6%)	
Location	Perimembranous	34/115 (29.6%)	46/115 (40.0%)	35/115 (30.4%)	0.007*
	Muscular	0/20 (0%)	11/20 (55.0%)	9/20 (45.0%)	
	Subpulmonary	2/3 (66.7%)	0 (0%)	1/3 (33.3%)	
	Multiple	2/4 (50.0%)	1/4 (25%)	1/4 (25%)	
Shunt direction	Left-to-right	39/105 (37.1%)	30/105 (28.6%)	36/105 (34.3%)	0.011*
	Right-to-left	4/14 (28.6%)	7/14 (50%)	3/14 (21.4%)	
	Bilateral	6/28 (21.4%)	17/28 (60.7%)	5/28 (17.9%)	
	Not detected	0 (0%)	7/11 (63.6%)	4/11 (36.4%)	
Maternal disease	No signs of disease	39/129 (30.2%)	50/129 (38.8%)	40/129 (31.0%)	0.350
	Influenza	5/13 (38.5%)	6/13 (46.2%)	2/13 (15.4%)	
	Hyperglycemia	6/14 (42.9%)	3/14 (21.4%)	5/14 (35.7%)	
	Hypertension	1/3 (33.3%)	0	2/3 (66.7%)	
	Hyperthyreosis	0/1 (0%)	1/1 (100%)	0/1 (0%)	

### ROC curve

**Fig. 6:**
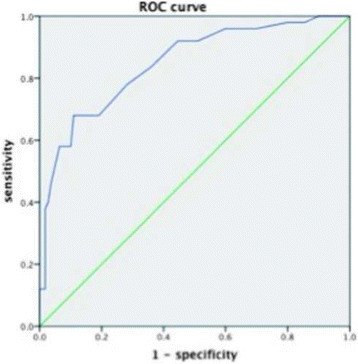
When SC is defined as a state variable and the defect diameter is defined as a test variable, the value of state variable is set to zero, and a receiver operating characteristic (ROC) *curve* can be achieved. Area under the curve is 0.842; *P* = 0.000; Upper cut-off value = 2.55 mm

When SC is defined as a state variable and the defect diameter is defined as a test variable, the value of state variable set to zero, a receiver operating characteristic curve can be achieved. Area under the curve is 0.842, *P* = 0.000. Cut-off value is 2.55 mm. In this case, sensitivity = 0.800, specificity = 0.718 (Fig. 6).

### Logistic regression analysis

To estimate the SC probability for a given defect diameter and birth weight, logistic regression analysis was used. After removing the impact of the defect location, birth weight was identified a protective factor [odds ratio (OR) value: 0.498; 95% CI 0.244, 0.978] while defect diameter was identified as a risk factor [OR value: 4.072; 95% CI: 1.885, 8.604] for SC. The relationship between defect diameter plus birth weight and SC probability was described by the following prediction formula: probability = (1 + exp -[-2.151-0.716*birth weight +1.393*diameter]) ^-1^.

### Verification of the prediction formula

**Fig. 7:**
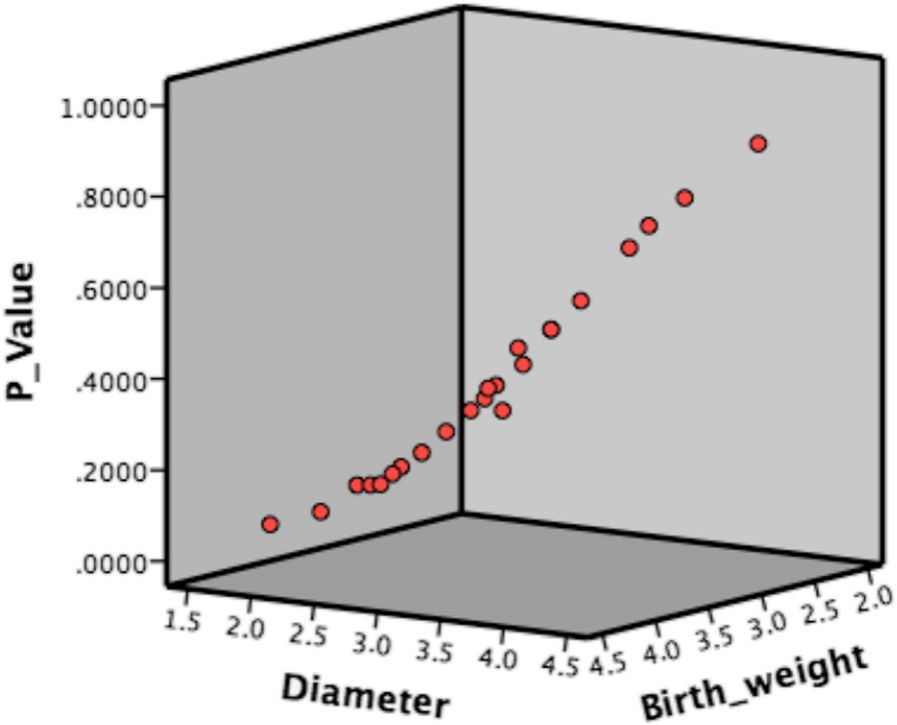
A three dimensional scatterplot was drawn by SPSS, diameter of the defect is X axis, *P* value is Y axis and birth weight is Z axis. As we can see, with lager defect diameter and lower birth weight come with higher *P* value, which means the defects are more likely to stay persistent

Follow-up outcome was compared with the probability calculated by our prediction formula, and 82.6% (19/23) of the predicted results coincided with the actual outcomes (Tables 3 and 4). We used these data to draw a 3 dimensional scatterplot, diameter of the defect is X axis, *P* value is Y axis and birth weight is Z axis. As we can see, with lager defect diameter and lower birth weight come with higher *P* value, which means the defects are more likely to stay persistent (Fig. 7).

**Table 3:** Verification: List of children whose defects spontaneously closed

ID	Predicted probability	Actual outcome	Time of SC	Birth weight (kg)	Defect diameter (mm)
01	0.2873	Closed	In gestation	3.1	2.7
02	0.3669	Closed	In gestation	3.4	2.9
03	0.1889	Closed	6 months	3.7	2.4
04	0.3133	Closed	3 months	3.15	2.6
05	0.1926	Closed	In gestation	4.25	2.7
06	0.1113	Closed	Neonatal	3.4	1.8
07	0.1454	Closed	3 months	3.75	2.2
08	0.2818	Closed	Neonatal	3.75	2.8
09	0.1033	Closed	Neonatal	4.1	2.1
10	0.2598	Closed	In gestation	4.1	2.9
11	0.5068	Closed	6 months	2.6	2.9
12	0.4700	Closed	In gestation	3	3
13	0.0619	Closed	In gestation	4.1	1.7
14	0.3049	Closed	6 month	3.4	2.7
15	0.1883	Closed	12 month	3.9	2.5

*SC* spontaneous closure

In the binary logistic regression, spontaneous closure is defined as 0, persisted defect is defined as 1, and predicted probability stands for the possibility of persisted defects

The predicted possibilities of the patients whose defects closed spontaneously are all below 0.5 (except for patent No.11)

**Table 4:** Verification: List of children whose defects didn’t spontaneously close

ID	Predicted probability	Actual outcome	Birth weight(kg)	Defect diameter(mm)	Note
01	0.8469	Thoracotomy surgery	2	3.8	
02	0.6264	Mortality	2.5	3.2	Respiratory failure
03	0.3467	Mortality	2.55	2.4	Down’s syndrome
04	0.4700	Defect reduced	3	3	
05	0.5496	Defect persisted	4.5	4	
06	0.7362	Defect persisted	3.15	3.9	
07	0.4056	Defect reduced	3.95	3.3	
08	0.7984	Defect persisted	3.05	4.1	

In the binary logistic regression, spontaneous closure is defined as 0, persisted defect is defined as 1, and predicted probability stands for the possibility of persisted defects

Patients with predicted possibility above 0.5 experienced surgery (No.01), mortality (No.02), or persistent defects (No.05,06.08), while patients with predicted possibility below 0.5 had reduced defects (No.04,07). Patient No.03 has Down’s syndrome

### Fetal karyotype of VSDs

Karyotyping was performed in 80 cases: 71 (88.75%) fetuses had a normal karyotype, and 9 (11.25%) cases of chromosomal anomalies were identified. Among the cases with aneuploidy, there were 7 cases with trisomy 18, 1 case with Turner syndrome, and 1 case with balanced translocations. Seven of 9 fetuses with trisomy 18 presented with a perimembranous VSD, 1 case with a mixed-type VSD and 1 case had a muscular defect (Table 5).

**Table 5:** Fetal karyotype of the VSD

Karyotype	Gestational weeks(w)	Maternal ages(y)	Location	Defect diameter	Outcome
47, XY, +18	26	26	Membranous	4.8 mm	TOP
46, XX, t(10;13)(p13;q14)	25	24	Membranous	1.6 mm	Closed
47, XX, +18	24	32	Mixed type	7 mm	TOP
45, X	26	28	Muscular	3 mm	TOP
47, XX, +18	24	32	Membranous	5.3 mm	TOP
47, XX, +18	23	43	Membranous	3.5 mm	TOP
47, XX, +18	22	30	Membranous	3.9 mm	TOP
47, XX, +18	22	25	Membranous	4.4 mm	TOP
47, XY, +18	26	29	Membranous	6.1 mm	TOP

*TOP* termination of pregnancy, *w* weeks, *y* years old

## Discussion

The present study was conducted in order to evaluate SC rate of isolated VSD and the clinical characteristics and prognosis of VSD patients from fetal age until 2 years after birth, in eastern China. While the incidence of VSD has increased in recent years, there has been advancement of fetal echocardiographic technology, allowing more accurate diagnosis of fetal congenital heart defects [31, 32]. Fetal heart examination is possible by transvaginal transducers as early as 9 − 10 weeks of gestation, and good quality transabdominal echo pictures are generally obtainable by 16 gestational weeks. In the current study, patients were evaluated during in second and early third trimester of pregnancy. Fetal cardiac screening is typically carried out at 18 − 22 gestational weeks, since fetal cardiac anatomical details, including ventriculoarterial connections, are possible to evaluate at this stage [33]. Subjects included in the present study were all in line with the indications for fetal echocardiography, ensuring efficient detection of CHD. Common referral indications include abnormal cardiac screening ultrasound, extracardiac anomalies shown on screening ultrasound, maternal diabetes, and family history of CHD [34].

In 1918, a French study reported the clinical findings in a young boy whose murmur and thrill disappeared at 5 years of age, documenting from first record of SC of VSD [35]. Here, we have reviewed reports on SC of VSD 1987 to 2015 from various countries, which have been detailed in Table 6. Reported closure rates vary from 6 to 88.9% with size and location of VSD, age at presentation and patient population, and results from our study are consistent with previous reports [6, 8, 9, 18]. SC of VSD is most common during the first year of life, with the likelihood of SC decreasing during adolescent years and adult life. It has been reported that the SC rate of patients with VSD not receiving surgical closure during childhood was as low as 6% [17].

**Table 6:** Single studies on spontaneous closure of ventricular septal defect

First Author	Place(Years)	Number	Starting point	Follow up	Classification	Classification of VSD	Spontaneous closure No.(%)	Influencing Factor
Moe [6]	Washington, USA (1987)	222	after birth	12 m	VSD	perimembranous 66 (65%), muscular 32 (32%), subpulmonic 3 (3%).	101 (48%)	location
Hornberger [7]	California,USA (1989)	66	6 m	40 m	VSD with CHF	perimembranous 45, muscular 19, supracristal 2	12 (18%)	size
Trowitzsch [8]	Datteln, Germany (1990)	169	<4 w	29 m		perimembraneous 35 (20.1%), muscular125 (71.8%), malalignment 12 (6.9%), subpulmonary 2 (1.1%)	70 (42.6%)	location
			4w ~ 1y			perimembraneous 32 (33.6%), muscular 57 (60%), malalignment 6 (6.3%)		
Frontera [9]	Valencia,Spain (1992)	882	>1y	9.5y		according toQp/Qs	275 (40.2%)	Qp/Qs
Ramaciotti [10],	Pennsylvania,USA (1995)	125	2.4 m	32 m	Muscular VSD	midmuscular 55 (44%), apical 31 (25%), anterior 33 (26%), and posterior 6 (5%)	30 (31%)	
Roguin [11],	Nahariya, Israel (1995)	56	6–170 h	10 m	Muscular VSD		40 (88.9%)	a delayed normal process
Du [12],	Nahariya, Israel (1996)	9/159	2–120 h	10 m	Muscular VSD preterm neonate		8 (87.5%)	
Shirali [13],	Texas,USA (1995)	149	<6 m	28 m		periembranous 100 (68.9%) muscular 49 (33.7%) canal-type 5 (3.4%) conal-septal 2 (1.8%)	46 (31%)	size and location
Krovetz [14]	Florida,USA (1997)	692	7 h–23y	3 ~ 18y			490 (70.8%)	
Turner [15]	Newcastle,UK (1999)	68	2d–42 m	76 m		small 49, moderate 14, large 5.	35.0%	location
Paladini [16]	Naples,Italy (2000)	68	24.8w	1y	Isolated VSD	Perimembranous inlet 22 (32.4%),outlet 19 (27.9%); muscular 7 (10.3%), malalignment 16 (23.5%)	In utero: 46.1%, postnatal : 23.1% not close: 30%	size and location
Gabriel [17],	Vienna, Austria (2002)	222	20.8y	7.4y		perimembranous 194 (84.8%), trabecular 30 (13.1%), outlet infracristal 4 (1.7%), inlet 1 (0.4%).	14 (6%)	Qp/Qs
Miyake [18]	Osakasayama, Japan (2004)	225	30d ~ 3 m	6y		perimembranous159 (70%) muscular 35 (16%) subpulmonary 31 (14%),	107 (48%)	location
Atalay [19]	Ankara, Turkey (2005)	42	<6 m	2 ~ 10y	Small Apical Muscular VSD		24 (57.1%)	neonatal period
Fliedner [20]	Bonn, Germany (2006)	146	23.4w	1y	Isolated VSD	perimembranous 15 (10.3%) muscular 131 (89.7%): trabecular 116 (79.5%), outlet 12 (8.2%), inlet 3 (2%).	in utero: 32.7%, postnatal: 44.3% not close: 23.0%	location
Abbag [39]	Saudi Arabia (2006)	86	14 m	66.3 m		perimembranous 67.4%, muscular 19.8%, inlet 7% subarterial 5.8%.	16.30%	age, size
Atik [21]	São Paulo Brazil (2008)	155	8d	1–18Y	Small VSD	perimembranous 68 (36.3%) muscular 119 (63.6%)	48 (75%)	location
Miyake [22],	Osaka, Japan (2008)	48	2y	17.8y	Perimembranous VSD	according to Qp/Qs	11 (23%)	Qp/Qs
Chang [23]	Kaohsiung, Taiwan (2010)	66		1y	MuscularVSD	midmuscular 37, apical 24, anterior 5	54 (81.8%)	
Jin [24]	Shandong, China (2012)	96	24 ~ 40w	3y		Perimembranous 14, Muscular 52	in utero: 3%, postnatal: 83.3% not close: 3.9%	size and location
Sun [25]	Shanghai, China (2014)	1873	1 ~ 6y	23.8 m	Perimembranous VSD		343 (18.3%)	Age diamete, Diffuse shunt flow, aneurysmal tissue
Erol [26]	Istanbul, Turkey (2014)	76	23.1w	1y	Muscular VSD		in utero: 6.8%, postnatal: 75%, not close: 18.2%	size
Xu [27]	Changchun, China (2015)	425	1d ~ 6 m	5y		Perimembranous313 (73.65%) muscular 53 (12.47%) subarterial 39 (9.18%) mixed-type 20 (4.70%).	93 (21.8%)	size, location, Qp/Qs, membranous septal aneurysm

Congestive heart failure CHF

In line with many previous reports, perimembranous VSD was the most prevalent form of VSD, and muscular VSD had an obviously higher SC rate [8, 10–12, 15, 18, 20, 22]. However, the average diameter of muscular VSD was smaller than other types and none of the subjects required surgical intervention. The closure of muscular defects begins with septal muscle adherence, followed by obliteration of the defect by a fibrous tissue plug [36, 37]. In closure of membranous defects, the VSD is “covered” by tricuspid valve tissue [38, 39], Nir [39] also suggested that closure of membranous VSDs may begin in utero and the mechanism of closure was similar to that of postnatally.

The postnatal closure of the muscular septum has been hypothesized that ventricular septal closure may not be limited to the fourth and fifth postconceptive weeks, and may extend throughout pregnancy and into the postpartum period. In many cases, these muscular VSD may result from a delayed normal process rather than from disease [11]. Therefore, to avoid unnecessary anxiety, parents should be informed of this benign muscular ventricular septal defect whether it is identified by echocardiography intentionally or accidentally.

The incidence of subaterial VSD in the present study was comparable to the incidence in the West [6, 20, 29], but much lower than that reported in Japan [18]. Although this type of VSD seldom closed spontaneously, SC patients in our study had no prolapse of the aortic valve at the time of SC. We therefore postulate that SC probably occurred by growth of muscular septum surrounding VSD. Previous studies have reported higher SC rates in smaller compared to larger defects [13, 16, 26]. In our research, Group 3 whose defects closed during gestation has the lowest average defect diameter, indicating that smaller defects are associated with better the prognosis, with a defect diameter cut-off value determined at 2.55 mm, when the SC occurs.

Subjects with SC during gestation had the highest average birth weight, with relatively better outcomes, probably due to better blood circulation and nutrient status. Birth weight is affected by maternal and fetal genotype as well as environmental factors, and placental function is also an important factor. Levin [40] and Levy [41] reported that low birth weight infant and intrauterine growth retardation(IUGR) were more common in children with cardiovascular malformations. Levy [41] also pointed out that a hemodynamic change may explain lower birth weight by alterations of the fetal circulation. Low birth weight and prematurity and are known risks for mortality during surgery for CHD [42]. Lesions causing left to right shunt, pulmonary outflow tract obstruction or left ventricular outflow tract obstruction can severely affect intrauterine growth, resulting in small for gestational age fetuses [43]. Wei et al. [44] reported that a weight over 2.4 kg at the time of cardiovascular surgery was associated with lower rates of postoperative infections. Fisher et al. [45] concluded that infants with serious congenital heart disease (CHD) appear to be at increased risk for necrotizing enterocolitis (NEC). The incidence of NEC is significantly higher in very low birth weight neonates when CHD is present. We hypothesized that children with higher birth weight have a better hemodynamic and nutritional status as well as a stronger immunity to fight against infection. In this case, the growth and development of children with higher birth weight were less susceptible to be affected by infections. Further studies on fetal anthropometry and haemodynamics are necessary to provide insight into the relationship between cardiovascular malformations and low birth weight. In addition, reports of SC rates in utero are highly variable (3–46.1%), with average defects measuring 1.5–2 mm in size [16, 20, 26, 28]. In the present research, this compared with 19.1% SC rate in utero.

VSDs can increase the risk of aneuploidy of the affected fetus and fetal karyotyping should be offered. Since not all cases in our study received serum screening, control for this possibility with regards to iVSD closure has been reported by Lee et al. [46], who found that presence of an i-VSD before 24 weeks does increase the risk of fetal aneuploidy. Extensive examination for extracardiac anomalies should also be performed. Furthermore, studies have demonstrated gender-based differences in fetal cardiac diseases [47], and the SC rate of male patients was higher than female patients in the current investigation. However, the causes of gender-based differences in cardiac disease are still not completely clear and require further investigation.

There were a number of limitations in the present study. Only 76.7% of patients agreed to echocardiographic reexamination, which may have affected the SC rates. In addition, the subjects included in the evaluation were young children, and SC rates may increase with advancing age. Due to these limitations, the available evidence allows for crude estimates and only in children aged 2 years and under. Therefore, assessment of long-term prognosis, especially in children beyond this age, is imperative.

## Conclusion

In summary, birth weight and prenatal echocardiographic measurement of size and location of VSD enables the estimation of SC probability in individual patients. These data would be particularly informative for patients whose defect diameter indicates a high SC probability. Information from this study can be used in counseling of patients’ families by helping them prepare for the possible outcome of their child’s disease.

## Abbreviations

CHD: Congenital heart disease
CHF: Congestive heart failure
SC: Spontaneously closure
TOP: Termination of pregnancy
VSD: Ventricular septal defect
